# P-905. Post One-year Outcomes for Corticosteroid Use in Non-HIV Cryptococcal Meningoencephalitis

**DOI:** 10.1093/ofid/ofae631.1096

**Published:** 2025-01-29

**Authors:** Bickey Chang, Seher Anjum, Snow Joseph, Dima A Hammoud, Peter Williamson

**Affiliations:** NIAID, Bethesda, Maryland; NIAID, Bethesda, Maryland; NIMS, Bethesda, Maryland; NIH Clinical Center, Bethesda, MD; National Institute of Allergy and Infectious Disease, Bethesda, Maryland

## Abstract

**Background:**

Cryptococcal meningoencephalitis (CM) is a major cause of mortality in HIV/AIDS, transplant recipients and previously healthy individuals. In the latter, a post-infectious inflammatory response syndrome (PIIRS) has been noted to have a favorable response to corticosteroids although long-term responses have not been studied.

**Methods:**

In addition to standard antifungal therapy, 15 patients admitted with CM/PIIRS from 2015-2020 were treated with standard pulse – taper corticosteroid therapy (PCT) consisting of intravenous methylprednisolone 1 gm daily for 1 week followed by oral prednisone 1 mg/kg/d, tapered based on clinical and radiological response. One patient was excluded from the study due to death from an unrelated cause within 6 months of diagnosis. Comprehensive neurocognitive testing and MRI brain scans were performed one year after diagnosis to assess clinical status.

**Results:**

80% of patients were male, median age 51 years. Median time from antifungal treatment to steroid initiation was 6 weeks. Eleven of 14 patients were neuropsychologically evaluated using performance-based neurocognitive tests. A one-sample T- test showed that the cohort obtained significantly lower scores in memory, learning, language, and information processing/psychomotor speed test in comparison to a normative sample. Also, the mean memory, learning, psychomotor, and information processing speed scores were in the low average range. These findings suggest very mild cognitive deficits in this cohort one year post diagnosis. With the exception of one patient whose MRI brain showed worsened findings at the one-year mark due to a neuro-inflammatory flare, radiological findings suggested stability or improvement for the remaining 13 patients. CSF cultures remained negative.

**Conclusion:**

Neurocognitive testing performed in a small cohort of previously healthy patients with CM-PIIRS showed an absence of moderate or major neurocognitive deficits one year post diagnosis with minimal adverse effects and no reoccurrence of infection. Brain MRI findings were also stable or improved for the majority.

**Disclosures:**

**All Authors**: No reported disclosures

